# Characterizing Tropical Tree Species Growth Strategies: Learning from Inter-Individual Variability and Scale Invariance

**DOI:** 10.1371/journal.pone.0117028

**Published:** 2015-03-10

**Authors:** Jimmy Le Bec, Benoit Courbaud, Gilles Le Moguédec, Raphaël Pélissier

**Affiliations:** 1 IRD, UMR AMAP, Montpellier, France; 2 AgroParisTech, Paris, France; 3 IRSTEA, UR EM, Saint-Martin-d’Hères, France; 4 INRA, UMR AMAP, Montpellier, France; 5 French Institute of Pondicherry, Puducherry, Tamil Nadu, India; Albert-Ludwigs-Universitat Freiburg, GERMANY

## Abstract

Understanding how tropical tree species differ in their growth strategies is critical to predict forest dynamics and assess species coexistence. Although tree growth is highly variable in tropical forests, species maximum growth is often considered as a major axis synthesizing species strategies, with fast-growing pioneer and slow-growing shade tolerant species as emblematic representatives. We used a hierarchical linear mixed model and 21-years long tree diameter increment series in a monsoon forest of the Western Ghats, India, to characterize species growth strategies and question whether maximum growth summarizes these strategies. We quantified both species responses to biotic and abiotic factors and individual tree effects unexplained by these factors. Growth responses to competition and tree size appeared highly variable among species which led to reversals in performance ranking along those two gradients. However, species-specific responses largely overlapped due to large unexplained variability resulting mostly from inter-individual growth differences consistent over time. On average one-third of the variability captured by our model was explained by covariates. This emphasizes the high dimensionality of the tree growth process, i.e. the fact that trees differ in many dimensions (genetics, life history) influencing their growth response to environmental gradients, some being unmeasured or unmeasurable. In addition, intraspecific variability increased as a power function of species maximum growth partly as a result of higher absolute responses of fast-growing species to competition and tree size. However, covariates explained on average the same proportion of intraspecific variability for slow- and fast-growing species, which showed the same range of relative responses to competition and tree size. These results reflect a scale invariance of the growth process, underlining that slow- and fast-growing species exhibit the same range of growth strategies.

## Introduction

Identifying the sources of variability in tree growth is critical to assess how the diversity of growth strategies shapes long-term forest dynamics and impacts ecosystem services such as wood production [1,2] or carbon storage [3]. In unmanaged tropical forests, tree growth is a highly variable process, among and within species, so that the individual tree responses to external drivers often appear idiosyncratic [4,5]. Interspecific growth differences may however reflect contrasted performances of species to secure carbon along a competition gradient [6,7], an ontogenetic trajectory [8] or with respect to site characteristics [9]. For instance, pioneer and shade-tolerant species strongly differ in their maximum growth, sensitivity to light and ontogenic trajectory [10].

A number of species are recognized as inherently slow- or fast-growing. Slow-growing species are generally characterized by a high leaf mass per unit area (LMA), a low concentration in nitrogen [11] and Rubisco [12], and thus a low rate of photosynthetic activity [13]. Interestingly, these traits were also found related to species shade-tolerance and sensitivity to competition [14,15] so that in line with the paradigm of a universal ‘fast-slow’ plant economics spectrum [16], species inherent growth rate is expected to be a synthesizing axis of species growth strategies. It results in a trade-off in performance at high vs. low resource availability, meaning that species growing fast at high resource levels perform poorly at low resource levels (relatively to some other species) and vice versa. Competition thus favors the species the most adapted to the local level of resources, which leads to partition species along resource gradients, a pattern that significantly contributes to species diversity maintenance [17,18].

However, this paradigm that species strategies can be summarized along a single axis is considered as over-simplistic by several authors [19–21]. It may be difficult in natural conditions to disentangle species intrinsic differences from the effect of habitat variation so that species growth performance may hide other axes such as species ability to withstand competition. In addition, a variety of strategies are successful in plant communities reflecting not only habitat diversity but also the fact that intraspecific traits variability may modulate individual responses to external factors [16,22].

In the last decade, the classical trade-offs paradigm underlying the species niche partitioning theory has been revisited. In particular, Clark et *al*. [20,23] pointed towards the role of high-dimensional differences among species in maintaining high diversity of forests. According to these authors, such process-level variation resulting from many, often unknown causes, makes individual growth responses to be highly variable within a species, and thus species responses to largely overlap, even if they differ in average. They showed that accounting for intraspecific variation of demographic and growth processes in simulations of forest community dynamics can lead some individuals of less competitive species to outperform individuals of the more competitive species and thus modifies the conditions in which species coexist in the long term. These results naturally raise the question of whether the intraspecific variability can be considered as a strategy for some species to persist in highly diverse ecosystems [22,24,25].

Modeling tree growth helps understanding to what extent species have different growth strategies [10,26]. But in most studies on tropical tree growth, a large proportion of the observed intraspecific variability remains unexplained by the limited number of measurable predictive variables. A particular feature of species rich tropical forests put forward to explain the low predictive power of tree growth models is the high number of rare species that often prevent the use of species-specific approaches. Species grouping has then been frequently used to reduce the number of parameters in multi-species growth models. It allows rare species that often represent a large number of available observations, to be included in analyses [27] by inclusion within larger groups. Species grouping helps to highlight structuring ecological strategies at the community level [28], but represent a loss of information. If classical species groups such as pioneer or understory shade tolerant are easily identified, little information is available for intermediate species, whose categorization thus depends on a priori knowledge on their growth behavior [27] or on *ad hoc* statistical criterions [29]. This often leads to rather heterogeneous groups and to an underestimation of the diversity of growth strategies.

In the present study, our goal was to identify species growth strategy axes from series of tree diameter increments recorded annually with permanent dendrometer bands over a 21-y period. Our dataset was comprised of c. 3,800 individual trees with stem girth ≥ 30 cm of 102 species in a permanent tropical forest sample plot in the Western Ghats of India. We addressed more specifically the following questions: (i) To what extent do species differ in their average response to competition, ontogeny and local abiotic environment? (ii) How might intraspecific variability help understanding species growth strategies? (iii) Is this variability consistent with the major proxy for tree growth strategies that represents species maximum growth rate? We chose a maximum likelihood hierarchical modeling approach [30] to deal with these different aspects in a single model thanks to the inclusion of random effects in addition to the fixed effects of covariates. In particular, random effects allowed us to include the growth responses of all species with a limited number of parameters, and to properly address the intraspecific variability in the growth responses, as well as the temporal autocorrelation of individual growth series. We then inferred species strategies based on a comparison of the species growth responses and their variability as captured by the mixed effect model.

## Materials and Methods

### Study Site and Data

Uppangala Permanent Sample Plot (UPSP; 12° 32' 15'' N, 75° 39' 46 E) is located at an elevation of 400–600 m a.s.l. in an undisturbed wet evergreen monsoon forest of the Pushpagiri Wildlife Sanctuary in the Western Ghats of India (see a detailed presentation in [31]). Permit for conducting a research program at Uppangala PSP was delivered by the Government of India through a Memorandum Of Understanding between the French Institute of Pondicherry (IFP) and Karnataka Forest Department (KFD) located in Bangalore, Karnataka state, India. The site is part of Kadamakal Reserve Forest, which comes under the *Dipterocarpus indicus*—*Kingiodendron pinnatum*—*Humboldtia brunonis* type of the low elevation dense moist evergreen forests of the region [32]. The climate is warm throughout the year (mean annual temperature of c. 27°C) and rainfall of about 5100 mm.yr^−1^, mainly from the Indian southwest monsoon, is concentrated between June and October (c. 90%) and alternates with a dry season with 4 months with rainfall < 100 mm.

The sampling plots are located on a north-oriented escarpment of the Ghats (average slope of c. 30–35°) characterized by a strong East-West alternation of deep talwegs and flattened interfluve ridges that determines steep slopes locally > 45°. The sampling design consists of transects and plots totaling together 5.07 ha that sample the variation in slope (see Fig. 4 in [31]). In these plots, all the trees above 30 cm of girth at breast height (gbh) or above the buttresses if any, were mapped within 10 x 10 m elementary subplots, identified to species level (species nomenclature refers to the Herbarium of the French Institute of Pondicherry, HIFP, http://ifp.plantnet-project.org/), and fitted with permanent dendrometer bands allowing a theoretical precision of 0.2 mm on gbh measurements. In total 3,870 trees belonging to 102 species have been yearly surveyed for gbh increment between 1990 and 2013. All subplots were georeferenced and a Digital Elevation Model (DEM) was derived from slope measurements taken at each corner of the subplots.

We worked with annual increment in diameter at breast height, Δdbh, computed from all pairs of consecutive girth records for each individual. Measurements of trees that died or were recruited (i.e. that reached a gbh of 30 cm) during the census period were included. A few observations were discarded because of missing girth records or in cases of doubtful precision, for instance when a note in the database indicated that the dendrometer band was disturbed or replaced. As extreme growth values were suspected to result from large measurement errors, we also discarded the two-tailed extreme 0.05% of the distribution of diameter increments, i.e. values below-0.5 cm.yr^−1^ and above 3 cm.yr^−1^. As the proportion of observations discarded did not significantly differ among species (chi-square test; *P*-value > 0.9), we considered that data reduction did not bias initial data. In total 3.4% of the 64,881 growth records were excluded from our analysis. The final dataset represented 3,810 synchronous series of individual annual diameter increments (in cm.yr^−1^), 2 to 21 years long, with about 80% spanning more than 15 years.

### Growth Model Design

A particular feature of our dataset is that trees were measured annually over a period of time that exceeds 20 years, so that data are highly temporally autocorrelated. In addition, growth data within a given species are also expected to be correlated. In order to deal with the intrinsic non-independence of observations and to compare species growth responses we used a hierarchical mixed model with an individual tree random effect and a date random effect both nested in a species random effect [30], which can be summarized as:

$$\Delta {\mathrm{dbh}}_{\mathrm{ijt}}=x_{\mathrm{ijt}}\times a+z_{\mathrm{ijt}}\times \alpha_{i}+\gamma_{\mathrm{ij}}+\delta_{\mathrm{it}}+\varepsilon_{\mathrm{ijt}}$$ eqn 1

where Δdbh_ijt_ is the annual diameter increment computed for each individual tree *j* of species *i*, as the difference between its diameter at *t* and *t-1* divided by Δ*t* in days to account for the slightly variable census intervals (from 305 to 426 days). Design vectors **x**_ijt_ and **z**_ijt_ contain observed covariates related to the fixed and species random effects with associated parameters in vectors **a** and **α**_i_, respectively. Altogether, the term **x**_ijt*_ **a**+ **z**_ijt*_ **α**_i_ represents a linear combination of the covariates where parameters are species specific (species random effects on both the model intercept and the slope of covariates). This term represents the average growth response of species *i* given values of the covariates for tree *j* at time *t*. Species effects were considered as random effects (i.e. parameters treated as realizations of a stochastic process and drawn in a common distribution) to force rare species parameters to be in a realistic range [30,33]. The term γ_ij_ is the individual random effect (on the intercept) for tree *j* of species *i*, which represents how much the growth trajectory of that tree deviates consistently over time from species *i* average growth response. The term δ_it_ is the date random effect (on the intercept) for species *i* at time *t*, and accounts for the synchronous growth variation at time *t* of all individual trees of species *i*. Finally, ε_*ijt*_ is the residual growth for tree *j* of species *i* at time *t* (assumed i.i.d.).

Such a model form allowed us to account for some major known covariates explaining tree diameter increment (see next section) and to structure the observed variability remained unexplained through the random effects. The individual tree random effect on the model intercept, γ_ij_, accounted for the inter-census correlation of growth thus avoiding confusion with the covariates' effect. The date random effect on the model intercept, δ_it_, accounted for the intra-census correlation of growth within each species. Interspecific variability in the growth response was assessed through several species random effects, both on the model intercept and on the slopes of covariates (**α**_i_).

### Covariates Selection

Covariates were selected from a review of the literature, from our expertise and by comparing different models (maximum likelihood estimations) using Akaïke Information Criterion (AIC), Bayesian Information Criterion (BIC) and likelihood ratio tests [34,35]. In a first step, we searched for the most appropriate form (quantitative vs. qualitative coding, variable transformation) of each candidate covariate independently. We then conducted a backward selection procedure starting from the full model including the relevant covariates and their interactions in order to select the best subset of covariates, i.e. the one providing the highest reduction in AIC and BIC when compared to the full model. Statistical significance of the covariates was assessed through likelihood ratio test of the nested models [30]. Rather than a systematic search of the best model among all possible combinations, we restricted our comparison to a set of realistic models that led us to retain functions of tree size, local competition and topographic position (slope and aspects) as fixed covariates.

Radial tree growth is generally expected to follow a hump-shaped pattern with respect to tree size [8,36,37], that is to increase with diameter for small trees, i.e. while increasing their leaf area towards maturity [38], and to decrease with diameter for large trees as they become senescent. We found that among the many combinations of diameter at breast height variables (*dbh*) proposed to represent this hump-shaped trajectory [8,39], the well known combination of *dbh* and log(*dbh*) led to the lowest AIC and BIC in our case.

In order to keep our model easy to interpret we introduced a single index of local competition calculated as the sum of neighbors' basal area within a radius of 5, 10, 15 or 20 m as generally proposed in the literature to represent resource depletion by competitors [40–43]. Such a competition index was preferred over an illumination index, such as Dawkins’ code for instance, because it is much easier to update annually in order to reveal temporal changes in competition intensity. Both size-symmetric (e.g. considering all the neighbors) and size-asymmetric (e.g. considering only the larger neighbors) competition indices are generally considered as important to represent below- and above-ground competition [44,45]. In our case, symmetric and asymmetric indices appeared systematically highly correlated (*r* ≥ 0.8 depending on the neighborhood radius considered). We kept a single index of symmetric competition in our model, because it appeared largely less correlated to *dbh* (*r* ≥ −0.06) than an asymmetric index (*r* ≤ −0.23). We also considered the log-transformation of this index [26] and finally, based on lowest AIC and BIC, we retained the log of total basal area of all the neighbors within a 15 m radius.

Topography at the study site, which alternates flattened interfluve ridges with steep slopes and deep talwegs that correlate with variation in soil thickness and sun exposure, is recognized as a major source of environmental heterogeneity [46,47]. We thus extracted from the Digital Elevation Model local values of terrain slope and aspect that we further attached to each tree. We compared the original slope variable (in %) to several slope classes with different thresholds based on the percentiles of the distribution. The slope variable leading to the lowest AIC and BIC was a simple classification into steep and gentle slopes using a threshold of 50%. Similarly, we compared the original aspect variable (in degree) with sine and cosine transformations that respectively emphasize East-West vs. North-South oppositions in slope orientation. Among various combinations with the slope variable [48], we retained the sine transformation describing the East-West alternation of slopes based on lowest AIC and BIC. This moreover corroborates the main feature of the site topography [31].

The growth model we finally fitted to the data followed the general form of equation 1 with the following fixed (**x**_ijt *_ **a**) and random effects (**z**_ijt *_ **α**_i_) components:

$$\begin{array}{c} x_{\mathrm{ijt}}\times a={\left[\begin{array}{c} 1\\ slope_{ij}\\ \sin(aspect_{ij})\\ \log(dbh_{ijt-1})\\ dbh_{ijt-1}\\ \log(ba_{ijt-1})\\ \log(ba_{ijt-1})\times \log(dbh_{ijt-1})\\ \log(ba_{ijt-1})\times dbh_{ijt-1} \end{array}\right]}^{T}\times \left[\begin{array}{c} a_{1}\\ a_{2}\\ a_{3}\\ a_{4}\\ a_{5}\\ a_{6}\\ a_{7}\\ a_{8} \end{array}\right]\\ z_{\mathrm{ijt}}\times \alpha_{i}={\left[\begin{array}{c} 1\\ \sin(aspect_{ij})\\ \log(dbh_{ijt-1})\\ dbh_{ijt-1}\\ \log(ba_{ijt-1})\\ \log(ba_{ijt-1})\times \log(dbh_{ijt-1})\\ \log(ba_{ijt-1})\times dbh_{ijt-1} \end{array}\right]}^{T}\times \left[\begin{array}{c} \alpha_{1i}\\ \alpha_{2i}\\ \alpha_{3i}\\ \alpha_{4i}\\ \alpha_{5i}\\ \alpha_{6i}\\ \alpha_{7i} \end{array}\right] \end{array}$$

Subscripts *i*, *j* and *t* stand for species, individual trees and dates, respectively. The variable *slope* is the local terrain slope described as a categorical variable with two classes (‘gentle’ and ‘steep’, coded 0 and 1, respectively); *aspect* is local terrain aspect (in degrees); *ba* is the basal area of all neighbors in a radius of 15 m (in m².ha^−1^); and *dbh* is tree diameter at breast height (in cm). Random effects are assumed normally distributed with mean 0 and independent from each other.

We performed Maximum Likelihood Estimates of the model parameters using package lme4 [49] for R statistical software (version 2.15.1; [50]). Parameter estimates (including variances of the random effects) are given in supporting information (S1 Table), along with a plot of model residuals that appeared normally and homogeneously distributed (S1 Fig.).

### Analysis of Model Predictions

We analyzed model predictions at the species level of our hierarchical model. Species growth response to each covariate was assessed from model predictions at standardized conditions, i.e. with the other covariates fixed at their observed mean [26]. From the species growth responses, we defined as species signed sensitivity to covariates, the range of growth predicted along the observed covariate gradients. As the response to competition was modeled by a monotonic function, the signed sensitivity to competition was computed as the difference between diameter increments predicted at maximum and minimum competition intensity encountered by the species. Signed sensitivity to competition was generally negative because growth decreased while competition increased. When the species response was not modeled by a monotonic function, as for aspect and tree size, the range of predicted growth did not necessarily corresponded to the difference between diameter increments predicted at both ends of the gradient. For the hump-shaped response to tree size, the signed sensitivity corresponded to the maximum difference between one end and the optimum of the growth response curve, the sign indicating whether this difference corresponded to an increase (positive) or a decrease (negative) in growth with tree size. For aspect variable, the signed sensitivity corresponded to the difference between the growth responses on East- and West-oriented slopes. Species sensitivity to competition, tree size or aspect was defined as the absolute value of the signed sensitivity.

In order to assess the range of growth strategies encountered in the forest community, species sensitivity to covariates was considered with respect to independent species attributes, such as species maximum growth, maximum size or abundance. Species maximum growth and size were taken as the 95^th^ percentile of the species values observed at the study site [29,51]. Abundance was taken as the average number of trees per species observed over the period of survey.

### Partitioning the Relative Importance of Fixed and Random Effects

For community-level interpretations of species growth strategies, we also compared how the within species variances partitioned with respect to the model terms. We thus refer in the following to the variance in observed diameter increments of species *i*, *σ*_*i*_²(Δdbh_ijt_), as the *intraspecific variability*, which partitions into a part explained by the covariates, *σ*_*i*_²(**x**_ijt*_ **a** + **z**_ijt*_ **α**_i_), or *explained variability*, and a part unexplained by the covariates or *unexplained variability*. One part of the unexplained variability is captured either by the individual effect, *σ*_*i*_²(γ_ij_), or by the date effect, *σ*_*i*_²(δ_it_). The remaining unexplained variability is the *residual variability*, *σ*_*i*_²(ε_ijt_).

We further assessed for each species the proportion of observed variability that was captured by the model using an extension of the simple formulation of R² for mixed models [52,53]:

$$R_{i}^{2}=\frac{\sigma_{i}^{2}(x_{\mathrm{ijt}}\times a+z_{\mathrm{ijt}}\times \alpha_{i})+\sigma_{i}^{2}(\gamma_{\mathrm{ij}})+\sigma_{i}^{2}(\delta_{\mathrm{it}})}{\sigma_{i}^{2}(x_{\mathrm{ijt}}\times a+z_{\mathrm{ijt}}\times \alpha_{i})+\sigma_{i}^{2}(\gamma_{\mathrm{ij}})+\sigma_{i}^{2}(\delta_{\mathrm{it}})+\sigma_{i}^{2}(\varepsilon_{\mathrm{ijt}})}$$ eqn 2

Because our model included in addition to fixed effects of the covariates, individual tree and temporal random effects likely to capture the effects of unmeasured growth drivers, we assumed that model residuals mostly accounted for measurement errors and stochastic effects, such as transient attacks of pathogens or herbivores. We therefore assumed that covariates, individual and date random effects captured most of the growth variability related to niche differences among species. As a consequence, we relied on the growth variability captured by model predictions rather than to the observed variability to assess the species growth strategies with respect to mechanisms of niche differentiation. We thus used within species variance ratios, slightly modified from [52], to explore how the variability captured by the model partitioned with respect to the terms of the model. The equations below represent the parts of intraspecific variability captured by the model attributable respectively to the effects of covariates (Equation 3), to the individual random effect (Equation 4) and to the date random effect (Equation 5):

$$R_{i}^{2}(x_{\mathrm{ijt}}\times a+z_{\mathrm{ijt}}\times \alpha_{i})=\frac{\sigma_{i}^{2}(x_{\mathrm{ijt}}\times a+z_{\mathrm{ijt}}\times \alpha_{i})}{\sigma_{i}^{2}(x_{\mathrm{ijt}}\times a+z_{\mathrm{ijt}}\times \alpha_{i})+\sigma_{i}^{2}(\gamma_{\mathrm{ij}})+\sigma_{i}^{2}(\delta_{\mathrm{it}})}$$ eqn 3

$$R_{i}^{2}(\gamma_{\mathrm{ij}})=\frac{\sigma_{i}^{2}(\gamma_{\mathrm{ij}})}{\sigma_{i}^{2}(x_{\mathrm{ijt}}\times a+z_{\mathrm{ijt}}\times \alpha_{i})+\sigma_{i}^{2}(\gamma_{\mathrm{ij}})+\sigma_{i}^{2}(\delta_{\mathrm{it}})}$$ eqn 4

$$R_{i}^{2}(\delta_{\mathrm{it}})=\frac{\sigma_{i}^{2}(\delta_{\mathrm{it}})}{\sigma_{i}^{2}(x_{\mathrm{ijt}}\times a+z_{\mathrm{ijt}}\times \alpha_{i})+\sigma_{i}^{2}(\gamma_{\mathrm{ij}})+\sigma_{i}^{2}(\delta_{\mathrm{it}})}$$ eqn 5

Species level variability structure was then considered with respect to independent species attributes. However, because rare species are characterized by few observations, percentile estimations of some attributes such as maximum growth and maximum size maybe biased [54]. We therefore conservatively interpreted model predictions and random effects for species with more than 10 individuals only, despite the fact that rare species were included for parameter estimation in our mixed model.

## Results

### Growth Responses Vary among Species

**Fig 1:**
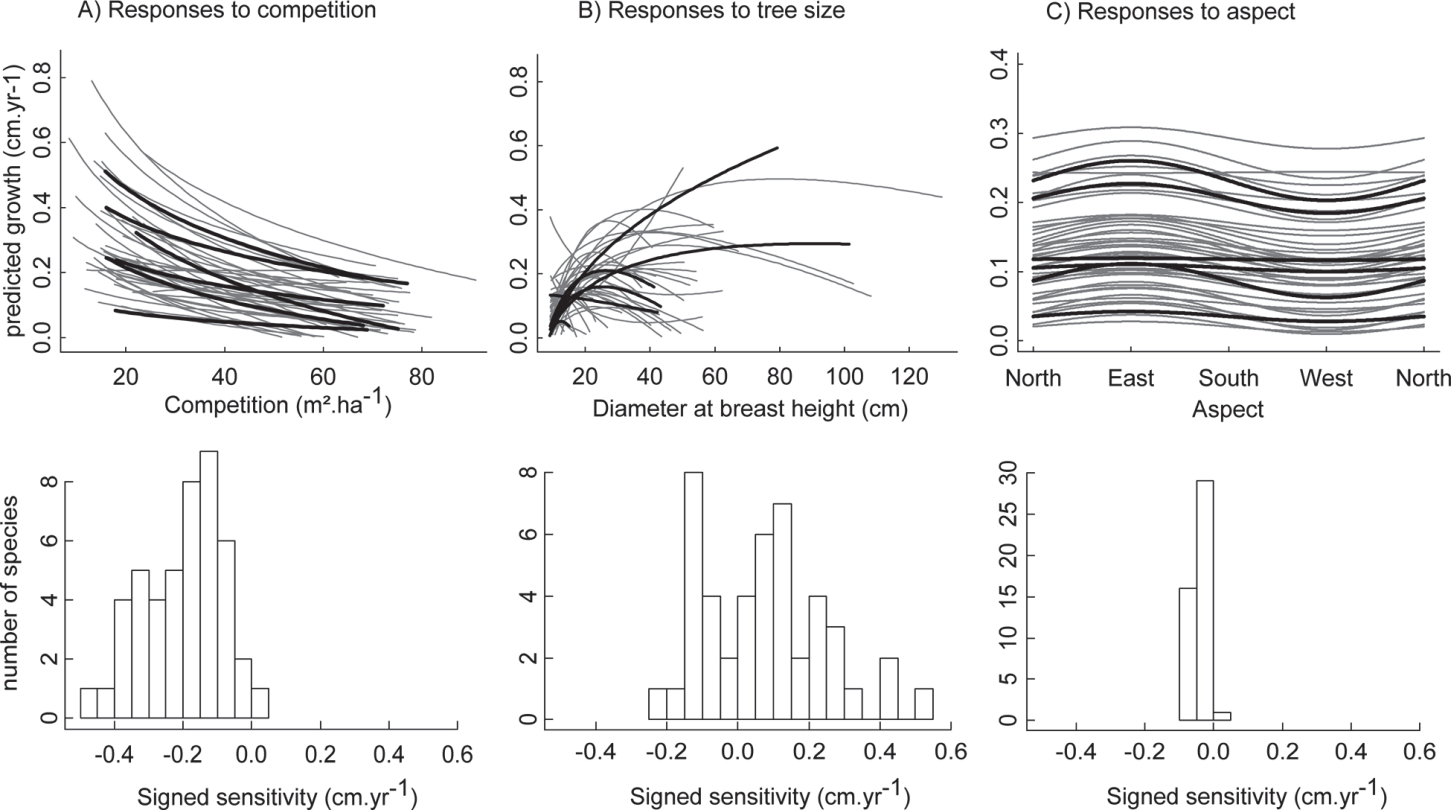
Predicted species growth response shapes and amplitudes to competition, tree size and aspect. Predicted growth at standardized conditions with respect to competition (A), tree size (B) and aspect (C), i.e. with the other covariates fixed at their observed means. The 6 most abundant species are in bold in top panels. Bottom panels represent the distribution of species signed sensitivity to covariates, as defined in section Analysis.

In standardized conditions, growth was predicted to decrease with an increase in local competition for most species (Fig. 1A), making signed sensitivity to competition to be negative. The pattern was more variable among species with respect to tree size and signed sensitivity to tree size was either negative or positive depending on which diameter species growth optimum was observed (Fig. 1B). Making aspect varying from 90° to 270° showed that most species grew faster on Eastern exposed hillsides (Fig. 1C). While a pronounced species growth rank reversal, exemplified by the crossing lines in Fig. 1B, was observed along the tree size gradient (Spearman's rho = 0.02 between predicted species growth at minimum and maximum observed tree size), rank reversal was moderate along the competition gradient (rho = 0.39) and almost inexistent along the aspect gradient (rho = 0.98). Tree size then appeared as a major axis species niche complementarity with respect to tree growth strategies.

**Fig 2:**
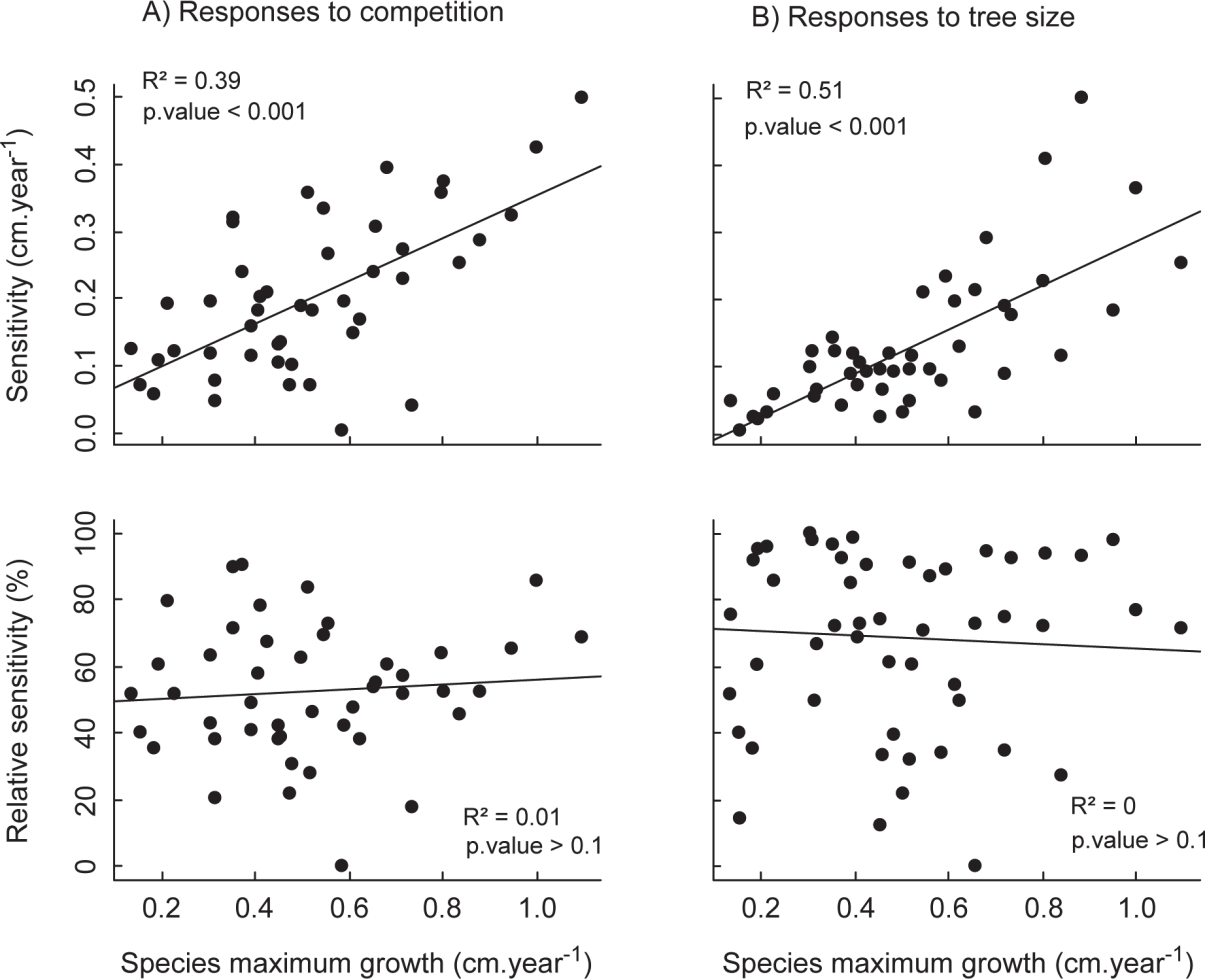
Species absolute and relative sensitivities to competition and tree size according to species maximum growth. Species growth sensitivity to competition (A) and tree size (B) with respect to species maximum growth. Sensitivity was estimated as the range of predicted diameter increments (as defined in Materials and Methods). In top panels, sensitivity is considered in absolute values (i.e., in cm.yr^−1^), while in bottom panels it is given in proportion of maximum predicted growth (in %).

Species growth sensitivity to competition and tree size increased significantly with species maximum growth (Fig. 2, top panels) and tree size (not shown). However, these trends vanished when considering relative sensitivity, i.e. sensitivity relatively to the maximum predicted growth (Fig. 2, bottom panels), suggesting that it reflected more a simple scale effect rather than an underlying functional relationship. Conversely, absolute and relative growth sensitivity to aspect appeared unrelated to species maximum growth and species maximum size. No relationship was found between species absolute or relative growth sensitivities and species abundance.

Note finally that slope affected similarly all species, with a mean diameter increment slightly lower on gentle slopes (−0.1 mm.yr^−1^) than on steep slopes, a pattern already documented at our study site [47].

### Intraspecific Variability Partitionning

**Fig 3:**
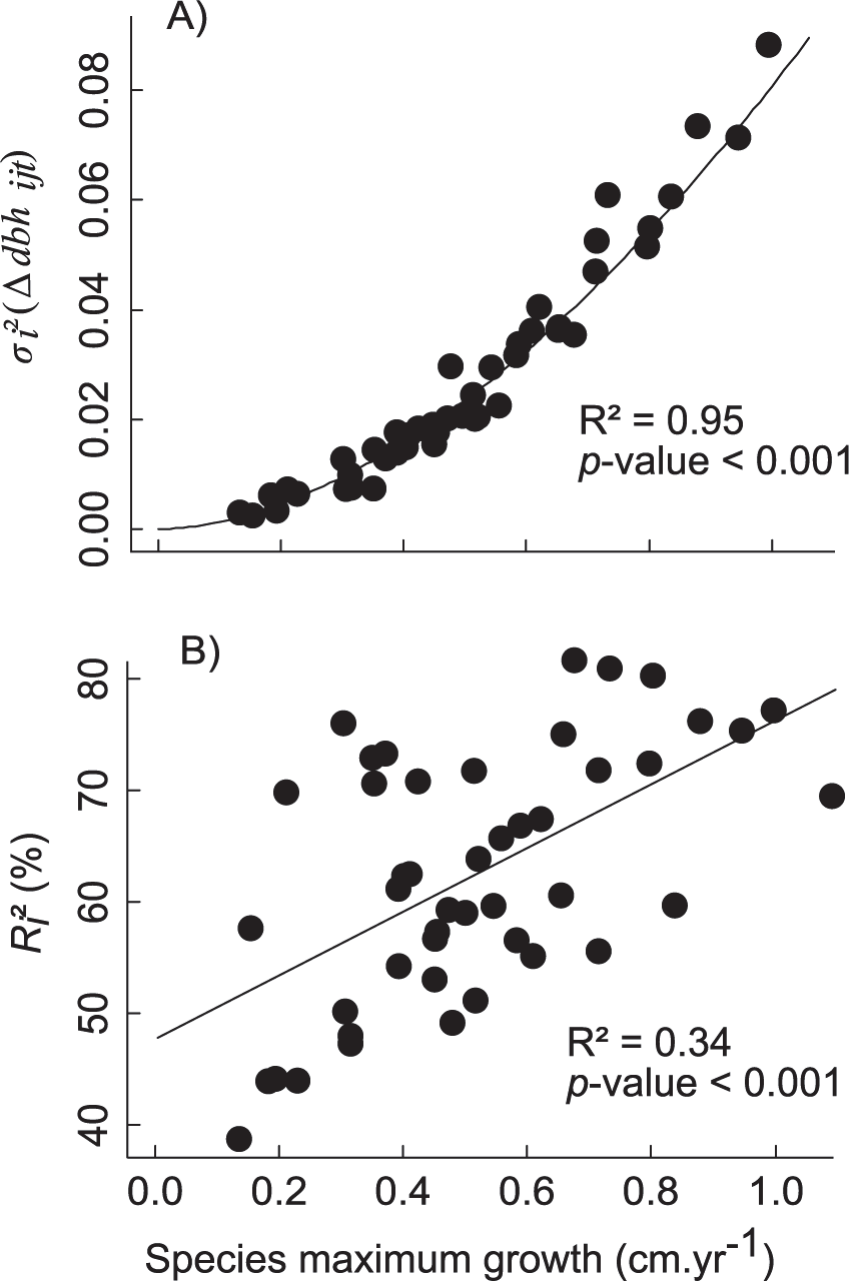
Observed and fitted growth variability according to species maximum growth. Variance of observed growth within each species according to species maximum growth (A).Proportion of this intraspecific variability captured by the model for each species (R_i_²) according to species maximum growth (B). The lines represent fitted relationships with a power (A) and a linear function (B).

Observed intraspecific growth variability, *σ*_*i*_²(Δdbh_ijt_), widely differed among species and increased significantly as a power function of species maximum growth (Fig. 3A). The proportion of this variability captured by the model (*R*_*i*_²) was on average 63% ± 11% (up to 80% for some fast-growing species) and also increased significantly with species maximum growth (Fig. 3B).

**Fig 4:**
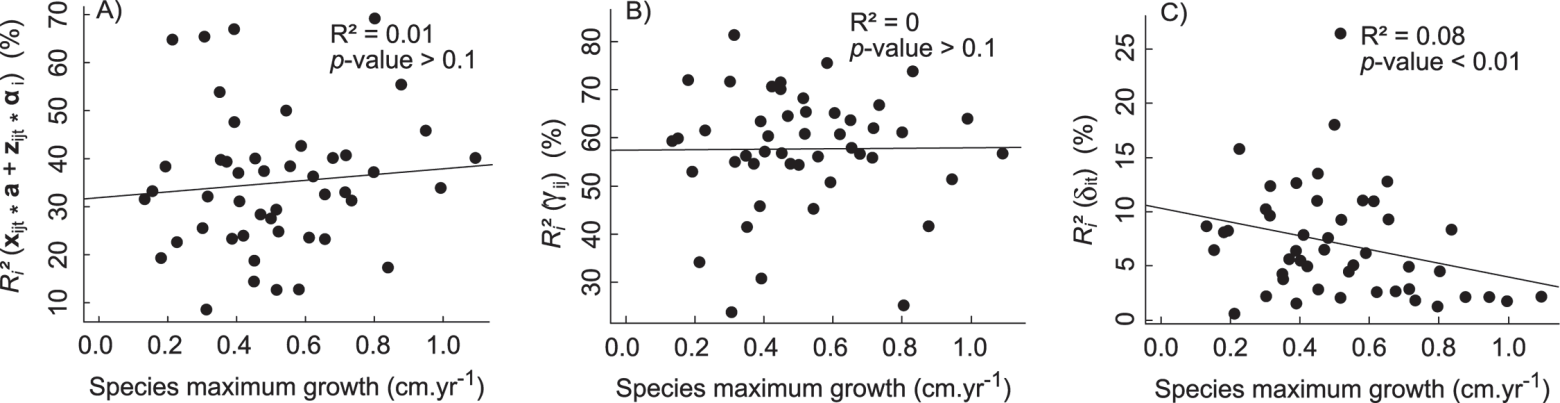
Fitted growth variability partitioning according to species maximum growth. Growth variability captured by the model with respect to species maximum growth. It is partitioned into explained variability (A), variability captured by the individual random effect (B) and variability captured by the date random effect (C).

Our hierarchical model allowed us to partition this captured variability within each species (see equation 1). On average one-third of it was explained by the covariates (*R*_*i*_^*2*^(**x**_ijt*_ **a** + **z**_ijt*_ **α**_i_) = 34% ± 13%), while more than half corresponded to the individual tree random effect, i.e. the inter-individual variability not explained by the covariates (*R*_*i*_^*2*^(γ_ij_) = 58% ± 11%), and a low proportion to the date random effect, i.e. the temporal variability unexplained by the covariates (*R*_*i*_^*2*^(δ_it_) = 7% ± 5%). The proportion explained by the covariates (Fig. 4A) or captured by the individual tree random effect (Fig. 4B) did not show any significant trend with species maximum growth, while the proportion of variability captured by the date random effect (Fig. 4C) slightly decreased with species maximum growth.

In addition, a one way analysis of variance performed for each species on the term (**x**_ijt*_ **a** + **z**_ijt*_ **α**_i_) with an individual indicator variable as factor revealed that 96% ± 3% of *σ*_*i*_²(**x**_ijt*_ **a** + **z**_ijt*_ **α**_i_), i.e. the intraspecific variability explained by the covariates, corresponded to inter-individual growth differences consistent over time. This proportion did not show any significant relationship with species maximum growth, maximum size or abundance. In other words, intraspecific variability in response to covariates was hardly explained by temporal fluctuations of the covariates. It follows that together with the individual tree and date random effects, inter-individual variability represents 87% ± 8% of the intraspecific variability captured by the model.

**Fig 5:**
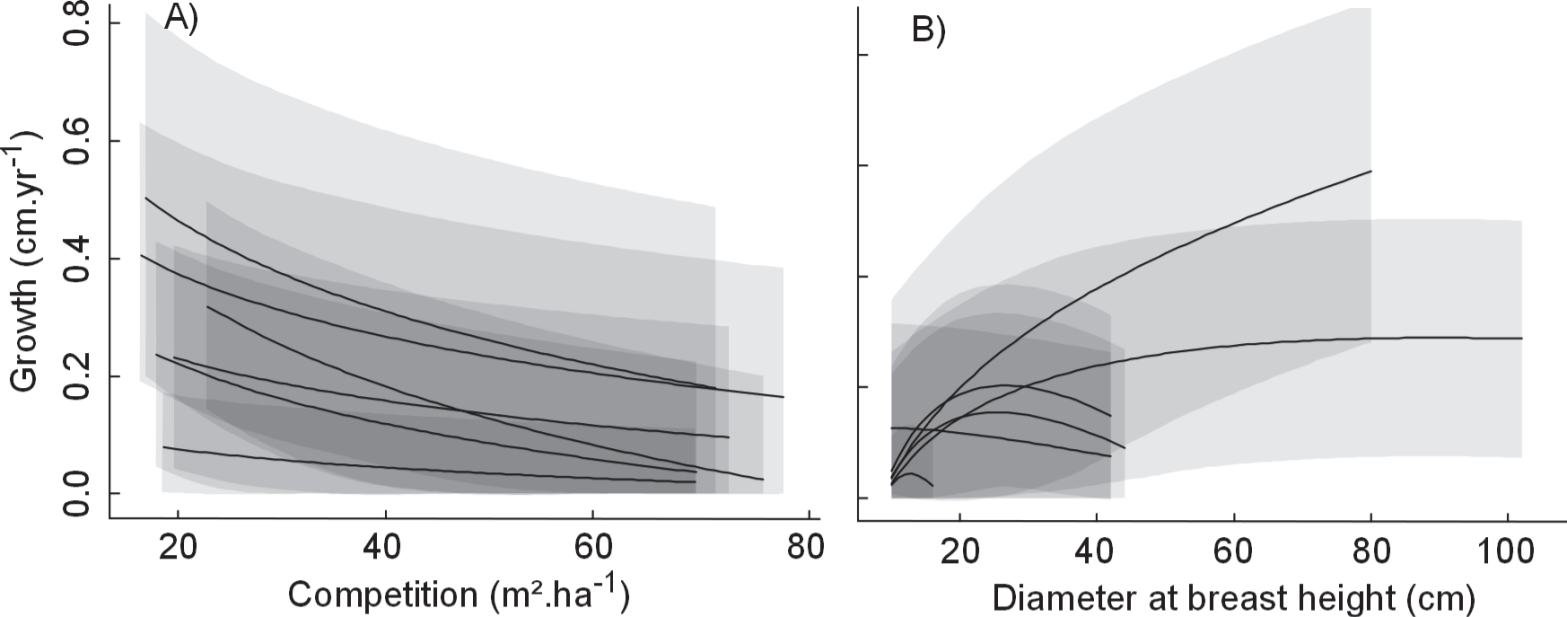
Species growth responses to competition and tree size overlap. Predicted growth response to competition (A) and predicted ontogenetic growth trajectories (B) at standardized conditions (i.e. with the other covariates fixed at their observed means) for the six most abundant species. Envelops represent the distribution of individual tree random effect (95^th^ percentile of the estimated distribution for each species).

The individual tree random effect quantified how each individual growth trajectory deviates from its species growth response, consistently over time and independently from the variation of covariates. Fig. 5 illustrates the distribution of the individual random effect around the predicted species response to competition and tree size for the 6 most abundant species at standardized conditions. It emphasizes that in spite of differences in growth predicted by the covariates, the large distribution of individual effects makes species responses largely overlapping.

## Discussion

### Species Show Highly Variable Growth Patterns

Nearly all species in our study showed a growth decrease with competition confirming that trees are sensitive to above- and/or below-ground resource depletion [26,55]. We also showed that species differed in their ability to sustain their growth when competition increases, leading to a moderate rank reversal in species performance along the competition gradient. These results suggest the existence of a trade-off between species growth rate and competition sensitivity [6,56] leading to potential resource partitioning among species [6,7,57]. Under strong competition, species with a high mean growth rate but sensitive to competition may be outperformed by species with a lower mean growth rate. Nevertheless, the fact that the observed rank reversal is moderate suggests that all the species might not follow this trend.

We also observed a continuum of ontogenetic growth trajectories and amplitudes with monotonically increasing or decreasing growth curves with diameter, but more generally hump-shaped growth trajectories. Such a pattern generally indicates that growth increases as trees progressively capture more light by reaching the canopy and spreading their crown [58]; then a decrease of growth with tree size is observed simply because a constant biomass investment leads to a smaller diameter increment in large trees, a pattern eventually enhanced by senescence mechanisms in late life-history stages. Our results revealed a strong rank reversal in growth performance along the tree size gradient. Such a pattern may result from ontogenetic niche shifts in some species [59], caused by physiological modifications with maturity and senescence in biomass allocation [60], photosynthetic traits [38] or mechanical constraints on water transport [61].

This rank reversal is however observed at standardized conditions, i.e. when competition is fixed at its observed mean. But the actual trajectory experienced by a tree may combine ontogenetic development and variations in competition environment. Our model underlines the importance of interactions between tree size and competition, since species response to competition changed with tree size and vice-versa. Such an interaction may have several origins. First, if below ground competition is likely to be size-symmetric, competition for light is expected to be size-asymmetric [62], so that a single symmetric competition index, used here to avoid overfitting issues, may underestimate the competition experienced by small trees. Second, due to ecological niche shift along ontogeny, some species may be more sensitive to competition at some particular stages. At last, species growth patterns may also be shaped by size-dependent and competition-dependent mortality. At high levels of competition, the selection pressure experienced in a population is high and slow growing individuals are likely to disappear rapidly [63]. It follows that the ontogenetic trajectory observed at the population level may be biased, expected to be monotonically increasing, with mature stages being represented only by highly performing individuals. Conversely, at low levels of competition, senescent individuals may survive longer, creating a hump-shaped ontogenetic population trajectory. Additional information on recruitment and mortality are therefore required to fully understand the pattern of interaction between tree size and competition on tree growth. Studying trade-offs between recruitment and mortality along environmental gradients could for instance greatly help understanding the importance of ecological niche shifts along ontogeny.

Our model predicted a higher diameter growth on steep slopes for all species, a pattern already documented at our study site [47] and interpreted as resulting from a better tree crown stratification on steep slopes providing a better crown illumination. Our model also revealed that growth was higher on East exposed hillsides first receiving sun light in the morning, but with an amplitude in the response that differed among species. It may be explained by the fact that photosynthesis could be more efficient during the morning [64] because lower temperature and higher air humidity limit evapotranspiration and allow stomata to stay open. The variable response of species to aspect might then reflect differences in species ability to maintain photosynthesis as the atmospheric conditions change. The combination of slope and aspect effects thus supports the thesis that light availability is an important driver of tropical tree growth [10,45].

### Individual Effects Improve Our Understanding of Species Growth Strategies

We showed that intraspecific variability explained by the model covariates corresponded mostly to inter-individual growth differences consistent over the 20 years of the study. This pattern results from the slow evolution of biotic growth drivers such as tree size and competition over the period. The low disturbance regime in Uppangala forests [46] might contribute to the stability of local competition and reinforce the fact that variability explained by covariates is mostly inter-individual, i.e. consistent in time. Covariates used in our analysis represent some of the major axes of niche differentiation among species. The fact that the variability explained by these covariates appears mostly inter-individual, i.e. is much higher than the intra-individual variability related to temporal variations of the covariates, confirms our hypothesis that niche related growth variability mostly reflects differences in growth performance between individuals of the same species, at least at the time scale of our study. In addition, by introducing an individual random effect in our model, we could take into account inter-individual growth differences unexplained by covariates. The fact that this individual effect still showed a structured spatial pattern (see S1 File) indicates that it also partly captured some unobserved heterogeneity of the growing conditions (e.g. soil texture, soil moisture or nutrient availability). Together, these two types of inter-individual differences captured a large proportion of the observed variability (almost 80% for fast-growing species). Thus, it strengthens our choice to analyze variability captured by the model rather than variability observed in the data.

In contrast, climate can be considered to induce synchronous temporal variations of growth within a population and a significant link exists between the date random effect in our model and regional inter-annual climatic variations (see S2 File). However, the date random effect accounted only for a low proportion of intraspecific variability (on average 7% of the variability captured by the model) suggesting that climatic variations did not strongly impacted tree growth compared to other growth drivers.

On average covariate effects, individual tree effects and date effects captured more than 60% of the observed variability. This proportion increased up to 80% for fast-growing species. A probable reason is that measurement errors or imprecision, which a priori do not dependent on species growth, mechanically account for a smaller proportion of the variability for species exhibiting higher variability, i.e. for fast-growing species. As a result, residual variability represents a higher proportion of the observed variability for slow-growing species. Thus, together with the fact that niche related growth variability is mostly inter-individual, it makes that focusing our analysis on the variability captured by the model (i.e. neglecting residuals) appeared as a reasonable hypothesis to study ecologically relevant (i.e. niche related) growth variability.

In spite of differences in species average growth responses and even changes in species growth ranking, we showed that species growth responses widely overlapped because of large individual tree effects. Inter-individual growth variability unexplained by covariates was about twice as large as the variability explained by covariates. The large growth response width evidenced here then suggests that the role of rank reversal in species coexistence should not be overemphasized [65]. Some studies have even reported no rank reversal in species rich tropical forests [26].

Inter-individual growth differences result from growth responses to identified covariates but probably also from many other unmeasured factors, including other environmental drivers and genetic heterogeneity within species or particular life history trajectory, as characterized by the notion of ‘personality’ in animal ecology [66]. This probable high-dimensionality of tree growth (i.e. high number of drivers of growth variability) is evidenced and advocated in recent contributions [19–21,23] as a key feature involved in species coexistence by promoting species niche complementarity along many gradients. Including inter-individual variability of growth performance in simulation studies is then promising to explore the conditions for species coexistence [25]. Unpredictable events (such as dispersion or mortality) are probably also involved in species coexistence [67] and should also be included in such studies. An underlying question is actually whether environment (biotic and abiotic) is really a determinant in explaining species coexistence (niche theory) or whether random events drive species coexistence (neutral theory) at local scale [67–69]. Our growth model constitutes the basis for simulation studies that would help disentangle stochastic and deterministic processes involved in species coexistence.

### Maximumgrowth Does Not Fully Determine Species Growth Strategies

We showed that, surprisingly, relative sensitivity to competition and tree size did not change with species maximum growth. Slow- and fast-growing species showed the same ability to increase or decrease their growth (in proportion) in response to competition change or along their ontogenetic trajectory. Congruously, we showed that the partitioning of variability captured by the model did not change with species maximum growth. These results suggest that growth is, to some extent, a scale invariant process which is perfectly in line with the power relationship found between intraspecific growth variability and species maximum growth [70]. Under this scaling effect hypothesis reflecting the mechanical increase of the variance with the intensity of a process (i.e. stability of the coefficient of variation), slow- and fast-growing species exhibit similar growth variation patterns, but at different scales (i.e. different intensity or different growth intensity).

These results challenge the classical idea of a continuum of growth strategies with slow-growing shade-tolerant species at one end and fast-growing pioneer species at the other end [28], summarized in the emerging ‘fast-slow’ paradigm of plant economics spectrum [16]. Under this paradigm, slow-growing species are in particular expected to be less sensitive to competition [10] or to drought [29] than fast-growing species.

We do not deny here that species inherent growth speed (characterized by their maximum growth in our study) is a key dimension of growth strategy. As it reflects the ability of species to reach maturity and to secure carbon, species maximum growth (growth scale) is a major axis to be taken into account in quantitative analysis such as carbon storage studies. Taking into account the relationship between the amplitude of species response to covariates (i.e. "sensitivity") and species maximum growth is then crucial but this relationship is rather the consequence of a scaling effect than the effect of differences in species growth strategies. Indeed, we showed that there was no relationship between species qualitative responses (or "relative sensitivity") to covariates and species maximum growth.

We suggest that in order to unambiguously compare species growth strategies in a qualitative way, species growth speed must be considered as a scaling factor to avoid confusion between growth strategy dimensions—also strongly recommended by Valladares et *al*. [71] regarding phenotypic plasticity studies. Using this approach, we showed that whatever species maximum growth, a continuum of growth strategies could be identified. We showed that maximum growth and responsiveness to competition or tree size were independent dimensions of species growth strategies. We believe that such scale invariance is not a particular feature of Uppangala forests but that it has been overlooked in other tropical forest study sites.

In our opinion, maximum growth, or more generally growth scale, should not be considered as a proxy for other growth strategy axes, but as an important axis, that should be taken into account when analyzing more refined variations among and within species.

## Supporting Information

S1 Fig Distribution of the model residuals. Standardized residuals of the growth model against fitted values of this model. The histogram represents the distribution of the standardized residuals.
(DOCX)

S1 File Spatial analysis of the individual random effect. (DOCX)

S2 File Testing for a climatic signal in the date random effect. (DOCX)

S1 Table Parameter estimates of the growth model. Estimated parameters for the fixed and random effects (σ stands for standard deviation).
(DOCX)
