# Supplementary material for: Characterizing Tropical Tree Species Growth Strategies: Learning from Inter-Individual Variability and Scale Invariance
Source: PLoS One. 2015 Mar 10;10(3):e0117028. doi: 10.1371/journal.pone.0117028 (PMC4355905; doi:10.1371/journal.pone.0117028)
Supplement: S1 Fig — Standardized residuals of the growth model against fitted values of this model. The histogram represents the distribution of the standardized residuals. (DOCX) [file pone.0117028.s001.docx]

**Figure S1:** Standardized residuals of the growth model against fitted values of this model. The histogram represents the distribution of the standardized residuals
