# Supplementary material for: Characterizing Tropical Tree Species Growth Strategies: Learning from Inter-Individual Variability and Scale Invariance
Source: PLoS One. 2015 Mar 10;10(3):e0117028. doi: 10.1371/journal.pone.0117028 (PMC4355905; doi:10.1371/journal.pone.0117028)
Supplement: S1 File — (DOCX) [file pone.0117028.s002.docx]

File S1: Spatial analysis of the individual random effect

# Context:

The growth model presented in our main document was spatially explicit because individual tree location was taken into account through the competition index and topographic variables. Nevertheless, given the rather simple covariates used with respect to the expected high-dimensionality of tree growth [1], it is likely that a spatial structured portion of the growth variability remained unexplained by our model. Because growth has been shown to be highly autocorrelated [1–3], most of the growth variability that may be informative and that is not explained by the covariates is expected to be structured within the individual effect. Figure 1 represents the spatial distribution of the individual random effect, which does not show any obvious large-scale spatial pattern. It reflects that the main factors of spatial correlation in growth were already accounted for in the growth model. However, as the result of latent (i.e. unobserved) heterogeneity of the growing conditions (mycorrhizae population dynamics, soil fertility, microclimate, etc.) more subtle correlation may still be hidden in this individual effect. Close individuals (in space) are in particular expected to be more similar in terms of individual effect than distant individuals because of shared spatially structured growth determinants. Such a spatial non-independence of individual effects could lead to reduced estimation efficiency and biased parameter estimation [4], especially the variance of the individual random effect [5,4,6]. We tested here the existence of a spatial correlation of the individual random effect using a semivariogram approach, commonly used to deal with spatial correlation in mixed modeling [5,4].

Figure 1 : Spatial distribution of individual tree random effect estimates. Each tree is represented by a point with size proportional to its individual random effect; red color indicates a negative effect, green color a positive effect.

# Method:

We used a semivariogram to quantify the degree of spatial dependence between the individual random effects (estimated from the growth model in the main document) of trees within the plots. The semivariogram is a mathematical function that has the following form [7]:

$\gamma\left( d \right)= \frac{1}{2}*E_{\sqrt{\left[ \left( x_{1}-x_{2} \right)^{2}+\left( y_{1}-y_{2} \right)^{2} \right]}=d} \left[ |Z\left( x_{1},y_{1} \right)-Z(x_{2},y_{2})|^{2} \right]$ Eqn. 1

where γ(*d*) is the semivariogram for the couples of observations distant of *d*. In a discrete setting, *d* represents distance classes. *E* is the mathematical mean function. Z(*x_1_,y_1_*) and Z(*x_2_,y_2_*) are the individual effects of two trees respectively positioned in (*x_1_,y_1_*) and (*x_2_,y_2_*). Random permutations of the individual effect values allowed computing the distribution of the semivariogram under the null hypothesis that no spatial correlation exists.

# Results:

Figure 2 shows that the observed semivariogram significantly deviated from the distribution (2.5th and 97.5th percentiles) of the semivariogram under the null hypothesis of spatial independence. It indicates that a significant trend exists in the spatial structure of the individual random effects of the growth model, with values more similar than expected between trees that are close from each other (i.e., less than 50 m apart) and more dissimilar than expected between trees c. 130 to 170 m apart.

Figure 2: Observed semivariogram of the individual random effect predicted from the growth model (red line) and 95% confidence envelop of the null hypothesis of spatial independence obtained from 1000 random permutations of the estimations among trees of the pattern.

# Discussion:

We evidenced here that individual random effects estimated by our growth model (see main document) were not spatially independent. Such a correlation, probably resulting from unobserved heterogeneity of the growing conditions (e.g. soil texture, soil moisture or nutrient availability), could have disappeared if appropriate additional covariates had been introduced in the model. There is no reason not to include such covariates when available. However, an extensive assessment of all the factors affecting growth is unrealistic as well as time and money consuming. As an alternative, introducing in the model an individual random effect ensures capturing and structuring a significant portion of the variability unexplained by the measured covariates [1]. Specifying a correct variance-covariance structure for the individual effect that would account for its spatial structure would be ideal. Different parametric functions have been proposed to model the semivariogram function (see for instance a quick overview of five classical semivariogram functions in [5]) but lme4 R package used to fit our growth model (initially chosen because it was one of the rare software allowing crossed random effects [8]) does not support yet this kind of models. The rising popularity of mixed models [8] and the dynamism of R software community is nevertheless promising. It is likely that available tools will improve in the next years and allow fitting more complex random structures to growing datasets for a better representation of the complexity of observed systems.

# References:

1. Clark JS, Bell D, Chu C, Courbaud B, Dietze M, et al. (2010) High-dimensional coexistence based on individual variation: a synthesis of evidence. Ecol Monogr 80: 569–608.

2. Swaine MD, Lieberman D, Putz FE (1987) The dynamics of tree populations in tropical forest: a review. J Trop Ecol 3: 359–366.

3. Brienen RJW, Zuidema PA, During HJ (2006) Autocorrelated growth of tropical forest trees: Unraveling patterns and quantifying consequences. For Ecol Manag 237: 179–190.

4. Fox JC, Bi H, Ades PK (2007) Spatial dependence and individual-tree growth models: II. Modelling spatial dependence. For Ecol Manag 245: 20–30.

5. Zuur AF, Ieno EN, Walker NJ, Savaliev AA, Smith GM (2009) Mixed effects models and extensions in ecology with R. New York: Springer.

6. Fox JC, Bi H, Ades PK (2007) Spatial dependence and individual-tree growth models: I Characterising spatial dependence. For Ecol Manag 245: 10–19.

7. Chauvet P (2008) Aide-mémoire de géostatistique linéaire. Paris: Presses des MINES.

8. Baayen RH, Davidson DJ, Bates DM (2008) Mixed-effects modeling with crossed random effects for subjects and items. J Mem Lang 59: 390–412.
