# Supplementary material for: Characterizing Tropical Tree Species Growth Strategies: Learning from Inter-Individual Variability and Scale Invariance
Source: PLoS One. 2015 Mar 10;10(3):e0117028. doi: 10.1371/journal.pone.0117028 (PMC4355905; doi:10.1371/journal.pone.0117028)
Supplement: S2 File — (DOCX) [file pone.0117028.s003.docx]

*File S2: Testing for a climatic signal in the date random effect*

# Context:

The growth model presented in the main document was based on repeated measurements of tree growth over a 21-year period. Even if this remains a rather short period to sample natural climatic variability and to identify potential long term effects of climate evolution (according to [1] at least 30 to 50 years are required), climate was expected to induce annual growth variability [2]. However, the relation between climate and tree growth is complex and potentially involves many interactions with other biotic or abiotic factors [3–5]. It may for instance have an effect that last (memory effect) or may trigger mast fruiting potentially leading to confusing climatically induced growth variations[4]. Climate may also affect differently above and below ground growth for the different species and affect interspecific competition. In addition, as generally advocated in [6] regarding tree growth, it is likely that the climate-growth relationship is high-dimensional.

In our modeling approach, we used a date random effect in order to account for all the growth variability that was synchronic among individuals (within each species), hypothesized to mainly reflect the effect of climate [2,7,8], and that was not explained by covariates However, given individual attributes (e.g. tree size, local competition, sun exposition) it is possible that trees respond differently to climate within species. In order to keep a reasonably simple model (i.e. avoid using multiple climatic covariates with multiple species random effects and interactions), we considered that most of the variability induced by climate was captured by the date effect (distribution in Figure 1). We showed in the main document that this effect accounted on average for 7% of the intraspecific variability.

Figure 1: Distribution (boxplots) and annual mean (red line) of the date random effects for 102 species.

In the climate change context, understanding the link between climate and forest dynamics is crucial. Thus we propose here a short discussion regarding both how the date effect is linked to climatic factors and how species may differ in their interannual growth variability patterns.

# Method:

We used a Principal Component Analysis with instrumental variables (PCAiv; [9]) in order to identify whether linear combinations of climatic variables could explain a part of species-specific date effect fluctuations. The PCAiv is a statistical tool used for data exploration that combines classical PCA and multivariate regression. A table containing the date effect of each species (columns) for each date (lines) as well as a table containing climatic variables for each date were used. Nine climatic variables were selected (Table 1): rainfall (proxy for the amount of water available for tree growth [10]), temperature (proxy for photosynthetic and respiration activity [11–14]) and SEPI (Standardized Precipitation Evapotranspiration Index [1]) which is a multiscalar drought index (based on the difference between precipitation and potential evapotranspiration) [13,15,16]. These climatic variables were aggregated over one year or taken only during January or July (preceding each census) respectively to reflect the average annual climate or the climate during the driest and the wettest month of the year [17].

Table 1: Climatic variables used in the PCAiv analysis (SPEI stands for Standardized Precipitation-Evapotranspiration Index)

| Climatic variable | Meaning | Data origin |
| --- | --- | --- |
| rainfall | rainfall over the last year | measured in Uppangala [15] |
| rainfall01 | rainfall from last January | measured in Uppangala [15] |
| rainfall07 | rainfall from last July | measured in Uppangala [15] |
| meanTemp | mean annual temperature | Climatic Research Unit [16] |
| meanTemp01 | mean temperature from last January | Climatic Research Unit [16] |
| meanTemp07 | mean temperature from last July | Climatic Research Unit [16] |
| spei | spei over the last year | http://sac.csic.es/spei/database.html [1] |
| spei01 | spei from last January | http://sac.csic.es/spei/database.html [1] |
| spei07 | spei from last July | http://sac.csic.es/spei/database.html [1] |

# Results:

Figure 1 highlights that the date random effect differed strongly among species but that it followed a general decreasing trend. On average, the date effect decreased markedly over the first period (1991-2001) and remained stable over the second period (1992-2011). The PCAiv allowed identifying the link between this trend and climatic variables. Figure 2 reveals that the first axis of the PCAiv captured most of the inertia within the date effect. It shows that mean temperature of January (corresponding to the driest month) was the climatic variable that was the most linked with the first axis of the PCAiv. More generally the three temperature variables are strongly correlated to this first axis. Annual SPEI (reflecting drought severity during the year preceding the growth measures) was the climatic variable that was the most correlated to the second axis of the PCAiv. In addition, Figure 2 shows that some specific date effects were rather related to the second axis than the first axis of the PCAiv. In particular it reveals contrasted patterns among the six most abundant species in the plot. The date effects for Vateria indica, Myristica dactyloïdes, Knema attenuata and Humboldtia brunonis were rather related to the first axis while for Palaquium ellipticum and Dipterocarpus indicus they were rather related to the second axis. The asymmetry of Figure 2B suggests that for all the species, to a certain extent, an increase of temperature is related to a decrease of the date random effects values.

Figure 3 confirms that the strength of the relationship between the first axis score of the PCAiv and the date effects for these same six species varied. Note that, even when the relationship is significant, the slope varied, reflecting a qualitative and quantitative variability in the temporal growth variability related to climate among species.

Figure 2: Correlation of climatic variables with the two first axes of the PCAiv on the species-specific date effects (A) and projection of species on the same axes (B). The six most abundant species are in red (B).

Finally, Figure 4 illustrates the particular case of the two most abundant emergent species (*Vateria indica* and *Dipterocarpus indica*) that exhibited an opposition of phase in their date effect (correlation between the two detrended date effects = -0.62).

# Discussion

Overall, this analysis revealed that the date effect did have a strong link with climate variability but that, as hypothesized, not a single climatic variable could explain these temporal growth fluctuations. Thus, we may conclude that the global trend observed in the date effect (decrease over the 20-year census period) largely resulted from climatic determinants but that directly including them in the growth model would have required many additional parameters and species random effects. Our results support findings in another study site in Costa Rica [14] that the decrease in growth observed at *La Selva* permanent tropical forest sample plot between 1984 and 2000 was related to the increase of atmospheric annual mean temperature (similar results also found in [18–20]).

Moreover, the discontinuous nature (two phases) of the date effect trend (Figure 1) together with the link with climatic variables, suggests that the date effect trend did not result from endogenous mechanisms. In particular this trend does not seem to result from the evolution of stand basal area because stand basal area (reflecting the level of competition among trees) changes at a slow continuous rate (i.e. did not exhibit two phases)^[[1]](#footnote-1)^.As a result, it means that the growth model correctly accounts for the effect of competition and reflects its temporal trend (endogenous to the stand dynamics).

Figure 3: Date random effect for the six most abundant species (ranked by abundance: Vateria indica, Humboldtia brunonis, Myristica dactyloïdes, Knema attenuata, Palaquium ellipticum and Dipterocarpus indicus) according to their score on the first axis of the PCAiv. Lines and p-values correspond to linear regressions.

Despite the identified link between climate and the date effect, it is however likely that temporal growth fluctuations captured by the date effect also result from biotic interactions. In particular, the example of opposition of phase between the two emergent species (*Vateria indica* and *Dipterocarpus indicus*) might result from an interaction between climate and species competitive ability. A scenario could be that when *Vateria indica* experience unfavorable climatic conditions, it benefits to *Dipterocarpus indicus* through a reduction of the competitive ability of *Vateria indica.*

Figure 4: Detrended random effect of the two most abundant emergent species Vateria indica (red) and Dipterocarpus indicus (green).

# References:

1. Vicente-Serrano SM, Beguería S, López-Moreno JI (2010) A multiscalar drought index sensitive to global warming: the standardized precipitation evapotranspiration index. J Clim 23: 1696–1718.

2. Clark DA, Clark DB (1994) Climate-induced annual variability in canopy tree growth in a costa Rican tropical rain forest. J Ecol 82: 865–872.

3. Kulam HM (1971) Effects of Insect Defoliation on Growth and Mortality of Trees. Annu Rev Entomol 16: 289–324.

4. Visser MD, Jongejans E, van Breugel M, Zuidema PA, Chen Y-Y, et al. (2011) Strict mast fruiting for a tropical dipterocarp tree: a demographic cost-benefit analysis of delayed reproduction and seed predation: Demographic cost-benefit analysis of masting. J Ecol 99: 1033–1044.

5. Ashton P, Givnish TJ, Appanah S (1988) Staggered flowering in the Dipterocarpaceae: New insights into floral induction and the evolution of mast fruiting in the Aseasonal tropics. Am Nat 132: 44–66.

6. Clark JS, Bell D, Chu C, Courbaud B, Dietze M, et al. (2010) High-dimensional coexistence based on individual variation: a synthesis of evidence. Ecol Monogr 80: 569–608.

7. Couralet C, Sterck FJ, Sass-Klaassen U, Van Acker J, Beeckman H (2010) Species-Specific Growth Responses to Climate Variations in Understory Trees of a Central African Rain Forest. Biotropica 42: 503–511.

8. Toledo M, Poorter L, Peña-Claros M, Alarcón A, Balcázar J, et al. (2011) Climate is a stronger driver of tree and forest growth rates than soil and disturbance: Climate drives tree and forest growth. J Ecol 99: 254–264.

9. Lebreton JD, Sabatier R, Banco G, Bacou AM (1991) Principal Component and Correspondence Analyses with Respect to Instrumental Variables : An Overview of Their Role in Studies of Structure - Activity and Species - Environment Relationships. Applied multivariate analysis in SAR and environmental studies. Eurocourses: Chemical and environmental science. Springer Netherlands, Vol. 2. pp. 85–114.

10. Worbes M (1999) Annual growth rings, rainfall-dependent growth and long-term growth patterns of tropical trees from the Aparo Forest Reserve in Venezuela. J Ecol 87: 391–403.

11. Lloyd J, Farquhar GD (2008) Effects of rising temperatures and [CO2] on the physiology of tropical forest trees. Philos Trans R Soc B Biol Sci 363: 1811–1817.

12. Clarke A, Fraser KPP (2004) Why does metabolism scale with temperature? Funct Ecol 18: 243–251.

13. Silva CE, Kellner JR, Clark DB, Clark DA (2013) Response of an old-growth tropical rainforest to transient high temperature and drought. Glob Change Biol 19: 3423–3434.

14. Clark DA, Piper S, Keeling C, Clark DB (2003) Tropical rain forest tree growth and atmospheric carbon dynamics linked to interannual temperature variation during 1984–2000. Proc Natl Acad Sci 100: 5852–5857.

15. Newbery DM, Lingenfelder M, Poltz KF, Ong RC, Ridsdale CE (2011) Growth responses of understorey trees to drought perturbation in tropical rainforest in Borneo. For Ecol Manag 262: 2095–2107.

16. Orwig D, Abrams MD (1997) Variation in Radial Growth Responses to Drought Among Species. Trees 11: 474–484.

17. Pélissier R, Pascal J-P, Ayyappan N, Ramesh BR, Aravajy S, et al. (2011) Tree demography in an undisturbed Dipterocarp permanent sample plot at Uppangala, Western Ghats of India. Ecol Arch 92: 17.

18. Asner GP, Townsend AR, Braswell BH (2000) Satellite observation of El Niño effects on Amazon Forest phenology and productivity. Geophys Res Lett 27: 981–984.

19. Braswell BH (1997) The Response of Global Terrestrial Ecosystems to Interannual Temperature Variability. Science 278: 870–873.

20. Sellers PJ, Los SO, Tucker CJ, Justice CO, Dazlich DA, et al. (1996) A Revised Land Surface Parameterization (SiB2) for Atmospheric GCMs. Part II The Generation of Global Fields of Terrestrial Biophysical Parameters from Satellite Data. J Clim 9: 706–737.

1. For instance, we evidenced that the stand basal area increased continuously by almost 10% (from 39.3m².ha^-1^ to 43.2m².ha^-1^) over the 21-y period. A miss-specification of the relationship between growth and competition within the fixed part of the model could have led to the temporal trend observed in the date random effect. [↑](#footnote-ref-1)
