# Supplementary material for: Characterizing Tropical Tree Species Growth Strategies: Learning from Inter-Individual Variability and Scale Invariance
Source: PLoS One. 2015 Mar 10;10(3):e0117028. doi: 10.1371/journal.pone.0117028 (PMC4355905; doi:10.1371/journal.pone.0117028)
Supplement: S1 Table — Estimated parameters for the fixed and random effects (σ stands for standard deviation). (DOCX) [file pone.0117028.s004.docx]

**Table S1:** Estimated parameters for the fixed and random effects (σ stands for standard deviation).

| Parameter | Estimate | Std. Error | t value |
| --- | --- | --- | --- |
| a_1_ | -2,0318 | 0,2248 | -9,04 |
| a_2_ | 0,0088 | 0,0042 | 2,07 |
| a_3_ | 0,0215 | 0,0050 | 4,26 |
| a_4_ | 1,3542 | 0,1078 | 12,56 |
| a_5_ | -0,0520 | 0,0056 | -9,36 |
| a_6_ | 0,4374 | 0,0583 | 7,51 |
| a_7_ | -0,2730 | 0,0276 | -9,89 |
| a_8_ | 0,0082 | 0,0015 | 5,36 |
| σ(α_1_) | 0.4658111 | - | - |
| σ(α_2_) | 0.0198642 | - | - |
| σ(α_3_) | 0.2770947 | - | - |
| σ(α_4_) | 0.0220096 | - | - |
| σ(α_5_) | 0.0480344 | - | - |
| σ(α_6_) | 0.0298305 | - | - |
| σ(α_7_) | 0.0067001 | - | - |
| σ(γ) | 0.1197966 | - | - |
| σ(δ) | 0.0448953 | - | - |
| σ(ε) | 0.1057442 | - | - |
